# A case study discovering lock-in effects of culinary culture and behaviours on cooking energy use in Chinese homes

**DOI:** 10.1038/s41598-026-35302-1

**Published:** 2026-01-29

**Authors:** Hong Wang, Han Lin, Saffa Riffat, Diran Yu, Yingqi Deng, Weixiang Wang

**Affiliations:** 1 Buro Happold Asia, No. 39 East 3rd Ring Road, Beijing, 100022 China; 2 AiGreen Energy Saving Technologies, Beijing, 100025 China; 3 Department of Architecture and Built Environment, University of Nottingham, Nottingham, NG7 2RD UK; 4 The Bartlett School of Sustainable Construction, University College London (UCL), London, WC1E 6BT UK; 5 China Academy of Building Research (CABR), No. 30 North Third Ring East Road, Beijing, 100013 China

**Keywords:** Cooking energy, Lock-in effect, Household energy, Family life cycle, Chinese culinary culture, Environmental sciences, Environmental social sciences

## Abstract

China’s rapid urbanization and industrialization have expanded building floorspace and contributed to rising carbon emissions in the building sector. An often-overlooked aspect of residential energy use is cooking, which this study examines through two longitudinal household case studies, combined with a questionnaire survey of 202 households. Monitoring results show that cooking accounted for 23% and 48% of total household energy consumption in the two cases, confirming its significant contribution to the residential carbon footprint. To further investigate the observed highly linear growth in cooking energy use in both households, a questionnaire survey was further conducted, revealing a lock-in effect correlating cooking energy with family life cycle (FLC) stages and habitual cooking practices, rather than with family size per se. To quantify this relationship, this study proposes a novel indicator, Cooking Energy Use Intensity (CookEUI), defined as the average daily cooking energy consumption (kWh/day). CookEUI ranges from 4.13 to 5.10 kWh/day for elderly and middle-aged couples, increases to approximately 6–7 kWh/day for two-generation households, and reaches 8.13–12.86 kWh/day for three-generation households with dependent children. Survey responses further indicate that cooking energy is strongly constrained by culturally embedded culinary behaviours. Our findings suggest potential solutions to reduce cooking energy use and emissions – such as alternative cooking appliances, cleaner energy sources, and community dining options – while respecting entrenched culinary culture, providing valuable insights for sustainable residential cooking practice and supporting efforts to reduce household carbon emissions.

**Supplementary Information:**

The online version contains supplementary material available at 10.1038/s41598-026-35302-1.

## Introduction

China’s trajectory of rapid economic growth over recent years has led to significant improvements in living conditions but has also escalated carbon emissions. In response, China has instituted dual-carbon goals to guide the transition toward low-carbon production and consumption^1^, which remains a core topic of China’s 15th Five-Year Plan towards 2030^2^. According to the ‌Research Report on Carbon Peaking and Carbon Neutrality in China’s Construction Industry^3^, the building and construction sector is the largest emitter, accounting for 39.1% of the country’s total energy-related carbon emissions, with residential buildings contributing approximately 60% during the operational phase. This underscores the residential sector’s significant role in achieving national dual-carbon targets.

Among various end-uses, cooking energy represents a unique but often neglected component. Per capita household cooking carbon emissions in China have shown a consistent upward trend over the past 30 years^4^, currently accounting for 19.1%, making it the second-largest residential energy consumer^5,6^. Comparative analyses further revealed that residential buildings in China had the lowest overall energy consumption compared to those in the United States and Japan, but cooking in China constitutes the highest proportion, at 35.2%^7^.

Despite this significance, cooking energy remains insufficiently studied. In conventional energy reports and building simulation software in China, cooking energy is typically categorised as “miscellaneous” alongside domestic hot water and plug loads, while major categories such as lighting, heating, cooling, and ventilation receive detailed attention^8–11^. This lack of disaggregation impedes efforts to mitigate high cooking energy consumption and hinders the development of efficiency strategies. Moreover, progress has been made in reducing building operational energy and achieving dual-carbon goals. The lack of a dedicated framework for studying cooking energy consumption, therefore, represents a critical research gap, both in empirical data availability and in the methodology of building performance analysis.

To address this gap, this study aims to delve into the cooking energy sector, characterising Cooking Energy Use Intensity (CookEUI) at the household level and exploring how it relates (conceptually and descriptively) to the Family Life Cycle (FLC). To achieve this, the research combines on-site measurements of cooking energy use in representative families, analyses household survey data to identify key behavioural and demographic factors and introduces a new quantitative metric to describe the discovery of unique cooking energy use patterns.

The novelty of this study lies in bridging technical energy measurements with social and behavioural dimensions to reveal the “lock-in effect” of cooking energy and the establishment of an innovative indicator specifically for cooking energy use. This interdisciplinary framework enables data-driven recommendations to improve cooking energy efficiency and sustainable building operation practices, while accounting for cultural factors.

## Literature review

China’s accelerated urbanisation and population growth demand urgent improvements in energy efficiency for new and existing residential buildings^12^. This study specifically targets the household cooking sector.

### Building cooking energy classification

Cooking energy in China lacks attention due to the commonly used energy classification methods. Considering China’s vast land area and diverse climate zones, the centralised heating (CH) system prevails in urban areas north of the “Qinling Mountain – Huaihe River” line^13^. TsingHua University Building Energy Research Centre (THUBERC) classified building energy consumption into four categories based on heating/non-heating energy and building types to reflect this diversity^14,15^. Similarly, most building standards and energy-related research have classified domestic energy without separating cooking energy^16–20^.

Some studies have examined building energy by energy types, but cooking, commonly using gas and electricity, is often grouped with hot water and other appliances due to metering limitations in Chinese buildings^21–25^. Other research has considered it as being too “user-behaviour dependent”^26^ or merely considered it less important to investigate^27,28^. In recent years, only a few studies have begun to address cooking energy in the Chinese context^29–31^, but they are either focused on traditional rural practices or lack quantitative and generalisable conclusions. In contrast, international research has increasingly explored cooking energy in both developed and developing countries^32–38^. This shows a research gap in systematic and quantitative research on Chinese cooking energy patterns.

Building energy simulations have revealed certain trends: for instance, cooking energy in Hong Kong residential buildings has risen steadily to about 20% of total household energy since 1984 ^39^, and has also shown a continuous increase in rural areas of mainland China^40^. Other analyses have also identified that cooking energy, combined with domestic hot water, is the largest carbon emission source apart from space heating and cooling^41^. Nevertheless, these analyses have not deeply explored the underlying causes beyond the general economic development and improved living standards. Multiple factors influence energy consumption dynamically and randomly, still demanding further investigation. Additionally, without in-depth investigation, existing standards may lack accuracy for setting constraint values for design, especially for cooking appliances, presenting a need for clearer guidance.

Recognising both the urgent national need to manage cooking-related energy and the global momentum of research in this field, this study addresses the existing knowledge gap through an innovative, bottom-up methodology. By using household-level field measurements and large-scale surveys, the study seeks to capture real-world energy use while accounting for behavioural uncertainty, thereby contributing new insights to sustainable building operation.

### Cooking culture evolution

China’s long and diverse culinary cultural background continues to influence contemporary cooking energy consumption and behaviours^42^. Over centuries, Chinese cooking methods have evolved across technologies, tools, cultural nuances, and regional preferences in cuisine. The prevailing cooking method shifted from boiling and steaming during the Tang and Song dynasties to the widespread adoption of roasting, smoking, stir-frying, and simmering by the Qing Dynasty^43–48^. Since the establishment of the People’s Republic of China, these traditional techniques have remained deeply embedded in daily life, while higher living standards have introduced new cooking appliances and methods, further diversifying energy use. For instance, a hasty cook can consume double the energy of a patient one^49^; pressure and rice soaking enhance rice cooking efficiency^50^, and steaming and simmering are notably more efficient than boiling^51^.

With recent economic development and urbanisation, China has experienced a major decline in biomass use^52,53^, and an increase in electrification, bringing substantial potential for electricity use for cooking in residential buildings^54,55^. However, this shift towards electrification has also led to public dissatisfaction, as Chinese culinary tradition, which devotes equal attention to the taste and aesthetic qualities of food as it does to aspects like nutrition, contrasts with Western culinary values^56–59^.

While China’s long and deeply rooted culinary culture shapes contemporary cooking behaviours, it also raises an important question: Is it realistic and feasible to expect meaningful cooking energy reductions through top-down behavioural interventions? A notable research gap exists concerning cooking practices in China, especially in urban contexts where electrification and appliance use are increasing. The intricacies of energy usage in Chinese cooking inspired this study to explore patterns of cooking energy variation across household types and the potential for change in real-life contexts, innovatively distinguishing family categories to identify underlying regularities and propose targeted strategies.

## Research methodology

Addressing the scarcity of domestic literature on household cooking energy, this study employs real-time measurement and surveys to gather authentic, first-hand data. The study was conducted from September 2021 to November 2022. Two families were selected for the household energy measurement study. One was a young couple in their 30s, and the other was a retired elderly couple in their 70s. Before starting to collect data, the participants were briefed on the purpose of the measurements, risks, and data disclosure, and gave informed consent to conduct the experiment. The conduct of this study was previously approved by the University of Nottingham Research Ethics Review Committee. The authors declare that all experiments were conducted in accordance with relevant guidelines and regulations. All participants gave informed consent to the study.

### Selection of test families for Real-Time measurement

Owing to the prohibitive costs of automatic instruments and data loggers, a group of families interested in understanding their cooking energy consumption and willing to manually record and provide daily data was chosen as the first step of the study. The chosen test families were representative, possessing the following characteristics.

The test families were located in Beijing. In Beijing, as the capital, living behaviours and cooking patterns are indicative of nationwide trends. Beijing’s buildings adhere to the highest national standards, and many systems within the building mandatorily include measuring devices^60^, facilitating energy consumption measurement. Meanwhile, Beijing’s representativeness of typical urban settings enables research results to guide national practices and serve as an international reference.

In addition to city selection, the chosen test families were representative in terms of income and family life cycle, regularly cooked at home, and commited to long-term data recording. In this case, families with children and elderly members were preferred, while solitary or newlywed couples who dined out frequently were unsuitable for the research^61^.

According to previous statistics^62^, the average apartment size in Beijing is 88 m^2^, typically ranging from 80 m^2^ to 95 m^2^ in floor area, and two bedrooms are the most preferred. Choosing families with this housing type ensures the representativeness of Beijing’s residential buildings for a typical assessment of cooking energy proportions.

### Cooking energy measurement

To understand the portion of cooking energy within the overall household energy consumption, plug-in meters were installed in all power sockets to measure the energy consumption of various home appliances, recording power in watts (W) and energy consumption in kilowatt-hours (kWh). A calorie meter and a gas meter were installed in the heating system and the cooking gas pipe. As household energy end users are neither complex nor difficult and expensive to monitor^63^, Option B of the International Performance Measurement and Verification Protocol (IPMVP) was employed. Readings of these meters were checked and recorded daily. Home appliance power use was categorised into seven groups: entertainment, cooking, lighting, heating, hot water, cooling, and others. The equations for major parameters calculation (related parameter explanation shown in Appendix B) are as follows.

$$\:{E}_{gas}=\:{V}_{gas}\times\:\:C{F}_{gas}$$

$$\:CookEUI\:=\:\frac{{E}_{cook},period}{{N}_{days}}$$

$$\:{E}_{annual}=\:{E}_{period}\times\:\:\left(\frac{365}{{N}_{days}}\right)\:$$

$$\:CookEU{I}_{annual}=\:CookEUI\:\times\:\:365$$

$$\:CO2e\:=\:su{m}_{i\in\:\left\{elec,gas\right\}}\left(\:{E}_{i}E{F}_{i}\right)$$

$$\:CO2{e}_{day}=\:{E}_{elec},day\:E{F}_{elec}+\:{V}_{gas},day\:E{F}_{gas}{m}^{3}$$

$$\:\:CO2{e}_{year}=\:CO2{e}_{day}\times\:\:365$$

### Radio frequency identification (RFID) for behavioural study

**Fig. 1:**
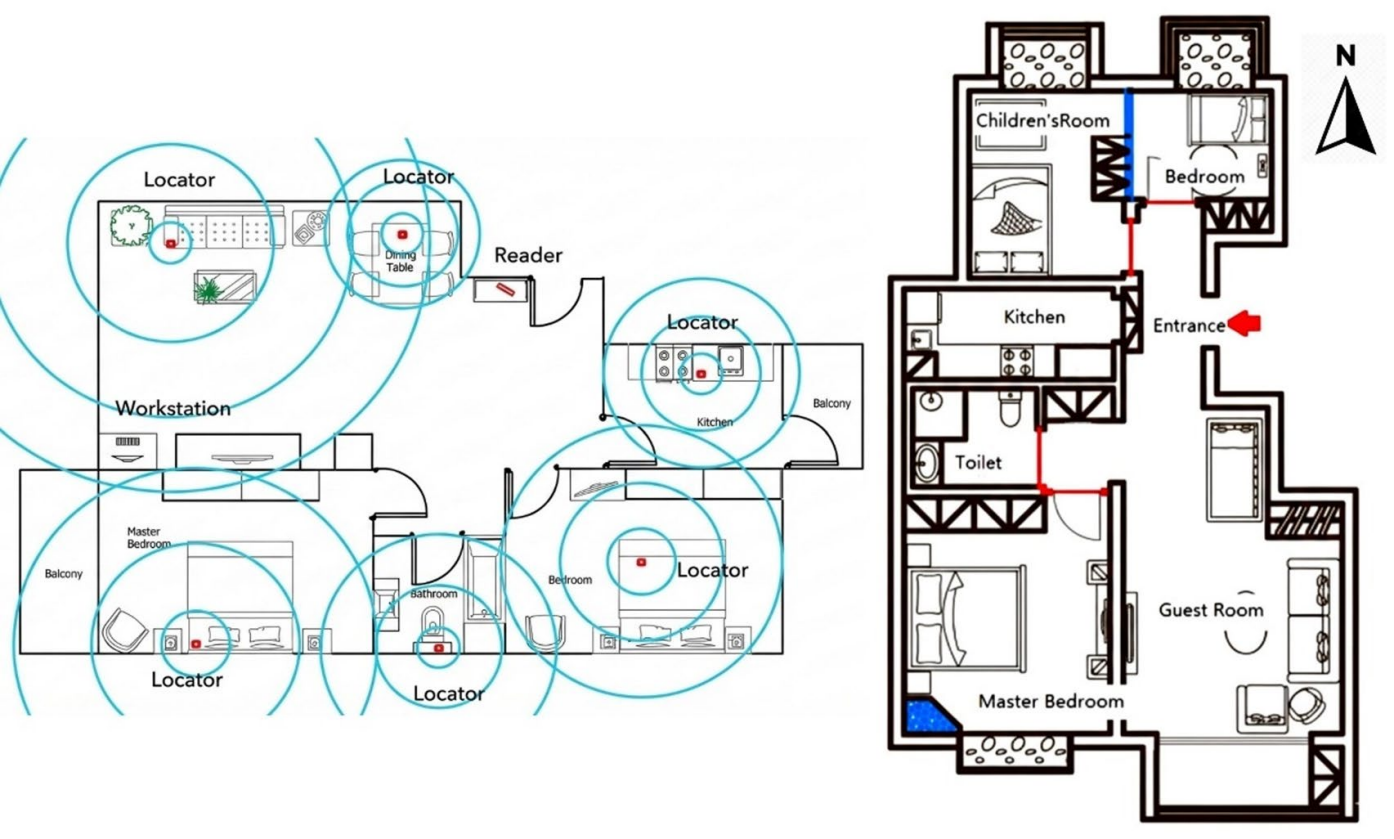
Floor plan of Test Family A with RFID system installed (left) and Test Family B (right).

Interviews and surveys to identify energy-efficient cooking practices are normally used in behavioural research. However, this approach may be subject to inaccuracies, especially with elderly participants, whose memories may become short and vague on many occasions. Therefore, for retired participants, an RFID real-time personal location system was installed. The system was comprised of a signal receiver/reader, personal locators, wristband tags, software, and a computer workstation. Personal locators in functional rooms triggered wristband tags upon entry, emitting signals that were received by the signal receiver (reader) and sent to the computer workstation. The software recorded room, person, and time data, facilitating later analysis of each family member’s time spent on cooking activities. Figure 1 (left) depicts the RFID system layout in the home of a retired couple.

### Cooking behaviour surveys

To concentrate on the relationship between cooking behaviour and energy consumption in later stages, the study also broadened its scope to include numerous secondary test families nationwide for a comprehensive sample. A survey was designed to encompass a wide variety of questions about family structure, family size, gas consumption, time of eating at home, cooking utensils, cooking methods, and food types.

Initially, the researchers distributed online surveys to a limited number of participating households, collecting responses for preliminary analysis. Subsequently, to refine the survey’s design to better align with the study’s specific objectives, adjustments were made based on the initial analysis findings. The revised questionnaire was then distributed to a larger, more diverse sample, enabling a more nuanced exploration of cooking behaviours on a national scale.

## Data collection

Two typical urban Chinese families were selected as in-depth case studies for household energy monitoring. The study was conducted over an extended period from late 2021 through 2022, allowing longitudinal observation of cooking practices and energy use. Prior to data collection, both households were informed of the study’s purpose and assured that the measurements were non-intrusive and were for research only, and informed consent was obtained.

Test Family A is an elderly retired couple in their 70s, residing in an 88 m^2^ unit on the third floor of a six-storey multi-rise residential building built in 2003. The unit comprises two bedrooms, one kitchen, one dining/guest room, and one bathroom.

Test Family B is a young couple in their 30s, their daughter, and her grandmother. This family lives in a 89 m^2^ unit on the sixth floor of a six-storey multi-rise residential building built in 2011. The unit comprises three bedrooms, one kitchen, one dining/guest room, and one bathroom.

### Household energy consumption and cooking behaviours

The measured data were manually recorded daily on a designated form. Electricity usage data were collected via plug-in meters, while space heating data were obtained from calorie meters installed in the heating pipe shaft. Gas usage, as recorded by the gas meter, was primarily for cooking purposes, given that both test families used electric water heaters for hot water. Table 1 shows the energy use of Test Family A and Test Family B. The data collection for Family A spanned from 21 November 2021 to 21 November 2022, while the data collection for Family B spanned from 13 September 2021 to 24 December 2021.

**Table 1:** Household energy use by category for test family A and B.

Home appliances		Whole year household energy use of Test Family A	3-month household energy use of Test Family B
Entertainment (kWh)	Computer	7.70	11.53
	Router		
	TV (bedroom)	231.42	110.97
	STB (bedroom)		
	VCD player		
	TV (dining/guest room)	275.06	
	STB (dining/guest room)		
Refrigerator (kWh)		249.32	73.43
Hot water (kWh)	Water heater (kitchen)	431.60	146.31
	Water heater (bathroom)	381.06	
Washing machine kWh		24.49	19.07
Cooling (kWh)	AC (bedroom)	28.62	27.97
	AC (dining/guest room)	0.00	
Cooking (kWh)	Fume exhaust fan	54.44	138.04
	Microwave oven	4.45	
	Electric rice cooker	116.98	
	Electric pressurised cooker		
	Soya milk maker	16.01	
	Fruit juice maker		
Other plug-in load (kWh)		300.35	176.88
Lighting (kWh)		50.50	125.40
Total electricity use (kWh)		2172.00	683.29
Space heating (kWh)		5815.90	946.26
Gas (m^3^)		220.10	137.58
Gas (kWh) (In Beijing, 1 m^3^ natural gas = 9.86 kWh^64^)		2170.19	1356.52

### Questionnaire survey

In addition to the in-depth field monitoring of the two primary test families, two rounds of questionnaire surveys were conducted with a broader sample of households. The goal was to explore whether the patterns observed in the case families reflect wider trends in cooking energy use and to examine potential relationships between cooking energy, occupant behaviour, and family type across a larger population.

The first survey round, launched in April 2023, was an online questionnaire covering topics such as family structure, family size, annual gas consumption, frequency of eating at home, cooking utensils and appliances used, preferred cooking methods, and typical food types. This initial survey collected 40 valid responses within two days, providing a preliminary dataset for analysis. After reviewing the results, the questionnaire was refined to better categorise family structure and capture relevant variables suggested by the initial findings.

To enhance the robustness and representativeness of the study, a second survey round was carried out with an expanded sample. This subsequent online survey gathered 162 valid responses from multiple provinces across China. The refined questionnaire again asked participants to report their household’s gas consumption (for the period from March 2022 to February 2023) alongside details of their family composition and cooking habits. For respondents who used piped gas for multiple purposes, additional information was requested to isolate the portion of gas used for cooking (excluding any gas used for water heating or other uses). In cases where participants indicated that their gas usage included water heating, we deducted an estimated hot water gas usage (based on typical usage patterns or any provided data) from the total to approximate the cooking-related gas consumption.

This broader survey data provided context for interpreting the case study findings. It allowed us to see how the two monitored families compared to other households in similar life cycle stages, and it helped identify general patterns linking family demographics and behaviours to cooking energy consumption.

## Experiment results and analysis

This section analyses the data collected, employing visualisations and introducing new concepts to unveil general patterns.

### Proportion of cooking energy consumption

**Fig. 2:**
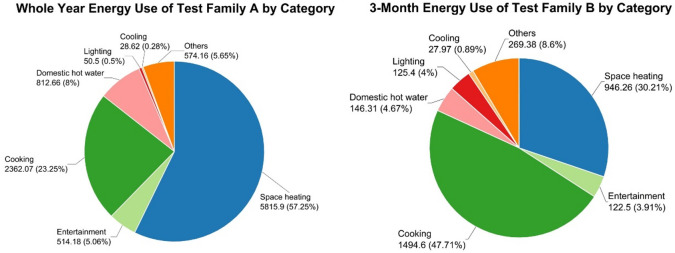
Energy Use Breakdown of Test Family A and B. (Note: Family A (~ 12 months) vs. Family B (~ 3.5 months). Cross-family comparisons should rely on kWh/day values in "Consumption patterns of cooking energy" section rather than totals.

In recording the energy consumption of Test Family A and B, the results (Fig. 2) reveal a significant insight that cooking, apart from heating, constitutes the most significant energy-consuming end use. This reaffirms the need for greater emphasis on cooking energy consumption compared to previous studies. As two longitudinal case reports, these results are descriptive and not statistically generalisable.

Quantitatively, cooking accounted for approximately 23% of Family A’s annual household energy and 48% of Family B’s quarterly household energy (Fig. 2). For cross-family interpretation, the average daily cooking energy was about 6.4 kWh/day for Family A and 14.7 kWh/day for Family B during their respective observation windows ("Consumption patterns of cooking energy" section). These values are descriptive daily averages; no inferential tests are conducted due to the case-report design. In both households, piped natural gas provided the majority of cooking energy, with electricity contributing a smaller share ("Consumption patterns of cooking energy" section). Because the observation windows differ (A ≈ 12 months; B ≈ 3.5 months), totals are not directly comparable; readers should rely on kWh/day values for cross-family comparison. These statements are descriptive and align with the paper’s aim of characterising household-level cooking energy use intensity rather than testing population-level hypotheses. Because observation windows differ, totals are not directly comparable; see "Consumption patterns of cooking energy" section for daily intensities.

### Consumption patterns of cooking energy

**Fig. 3:**
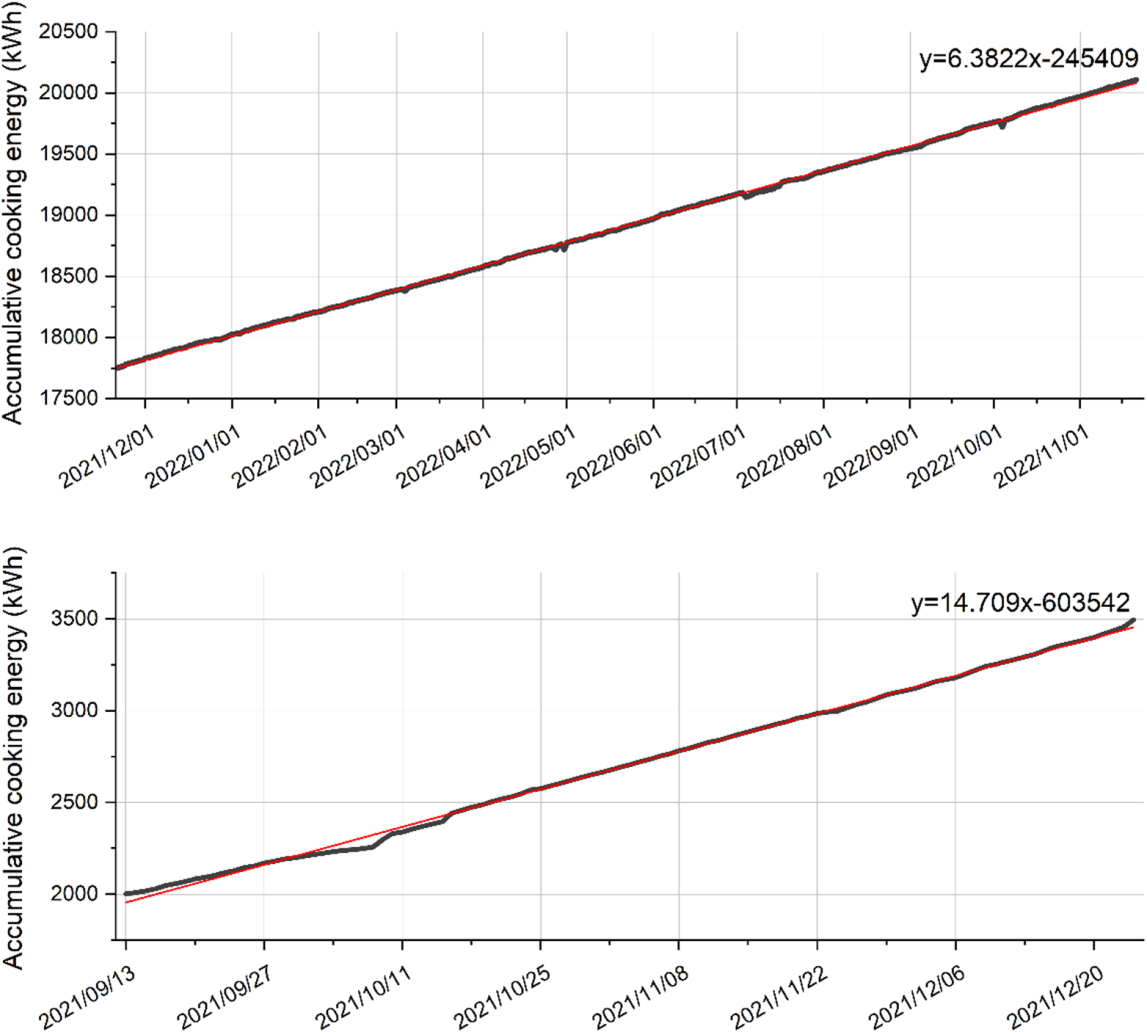
The accumulative cooking energy use of test Family A (top) and test Family B (bottom).

Beyond total shares, we examined the temporal pattern of cooking energy use in each family. Figure 3 illustrates the cumulative cooking energy consumption over time for Test Family A and Test Family B. Both households show roughly linear growth in cumulative energy use, indicating a relatively steady day-to-day cooking energy demand. However, the slopes of these trend lines differ markedly: Family A’s cooking energy accumulates at approximately 6.38 kWh per day, whereas Family B’s accumulative rate is about 14.71 kWh per day. These slopes (obtained from linear fits to the cumulative consumption data) effectively represent the average daily cooking energy consumption (CookEUI) for each family during the monitored period. Family A (elderly couple) used about 6.4 kWh of energy per day for cooking, while Family B (young three-generation family) used about 14.7 kWh/day. These values are descriptive daily averages; no inferential tests are conducted due to the case-report design.

This stark difference implies that Family B’s daily cooking energy usage is more than double that of Family A. Notably, Family B also has twice as many household members as Family A (four vs. two), but the higher CookEUI may not be explained by family size alone – lifestyle and cooking habits are also influential. For instance, Family B’s household likely prepares more dishes per meal (to cater to three generations’ tastes) and may cook more frequently (including for a young child), whereas Family A, as a retired couple, may cook fewer or simpler meals daily. The data from these two families prompted us to explore the concept of a “lock-in effect” between family characteristics and cooking energy consumption. The observation that a four-person family (Family B) consumed 14.7 kWh/day, while a two-person family (Family A) consumed 6.4 kWh/day, suggests that it might be the family structure and life stage, rather than just the sheer number of people, that influence cooking energy demand. In other words, different types of families appear to settle into different typical daily cooking energy levels.

Additionally, we investigated how much of each family’s cooking energy was provided by gas versus electricity. For both families, a majority of the cooking energy came from piped natural gas (used for stovetop cooking). Family A used about 220 m³ of gas over the year (approximately 5.95 kWh/day in heat content) and a smaller portion from electric cooking devices (e.g. rice cookers), whereas Family B used ~ 137.6 m³ of gas over approximately 3.5 months (roughly 13.2 kWh/day equivalent) plus some electricity for appliances like the exhaust fan. This indicates that gas-fuelled cooking (e.g. wok stir-frying and boiling) was the primary contributor to their cooking energy totals.

**Table 2:** Cooking energy use and CO_2_ emissions of test family A and B.

Family	Monitoring Period	Total Cooking Energy (kWh)	CO2 Emissions (kg CO2e)	Annualised CO2 (kg/year)
Test Family A (Elderly couple)	~ 12 months (Nov 2021 – Nov 2022)	2362 kWh (gas + electric)	~ 540 kg CO2 (12 months)	~ 540 kg/year
Test Family B (Young three-generation family)	~ 3.5 months (Sep – Dec 2021)	1495 kWh (gas + electric)	~ 350 kg CO2 (3.5 months)	~ 1240 kg/year*

Greenhouse Gas Emissions from Cooking Energy: To evaluate the carbon implications of the households’ cooking energy use, we converted the measured energy consumption into CO_2_ equivalent (CO_2e_) emissions using standard emission factors. Specifically, electricity consumption was multiplied by an emission factor of 0.61 kg CO_2_ per kWh and natural gas consumption by 1.94 kg CO_2_ per m³ (reflecting typical values for China’s energy supply). Using these factors, Family A’s cooking energy use (over ~ 12 months) corresponds to approximately 540 kg of CO_2_ emissions, while Family B’s cooking energy use (over ~ 3.5 months) corresponds to roughly 350 kg of CO_2_. If Family B’s cooking pattern were maintained for a full year, it would result in an estimated ~ 1240 kg CO_2_ per year from cooking, which is more than double Family A’s annual cooking emissions. Table 2 provides a summary of each family’s cooking energy consumption and the associated GHG emissions. These results highlight the significant carbon footprint that routine cooking activities can have and reinforce that households like Family B (with more intensive cooking routines driven by life cycle stage and cultural habits) can lock in higher emissions levels unless changes are made.

### Lock-in effect between family life cycle (FLC) and cooking energy

This study treats this as a conceptual, descriptive linkage rather than causal identification. During the survey of 40 samples conducted in the later stage of the study, it was discovered that different major categories of family compositions formed groups in terms of cooking energy consumption patterns. This also led to the hypothesis that there is a connection between family structure and cooking energy consumption.

**Fig. 4:**
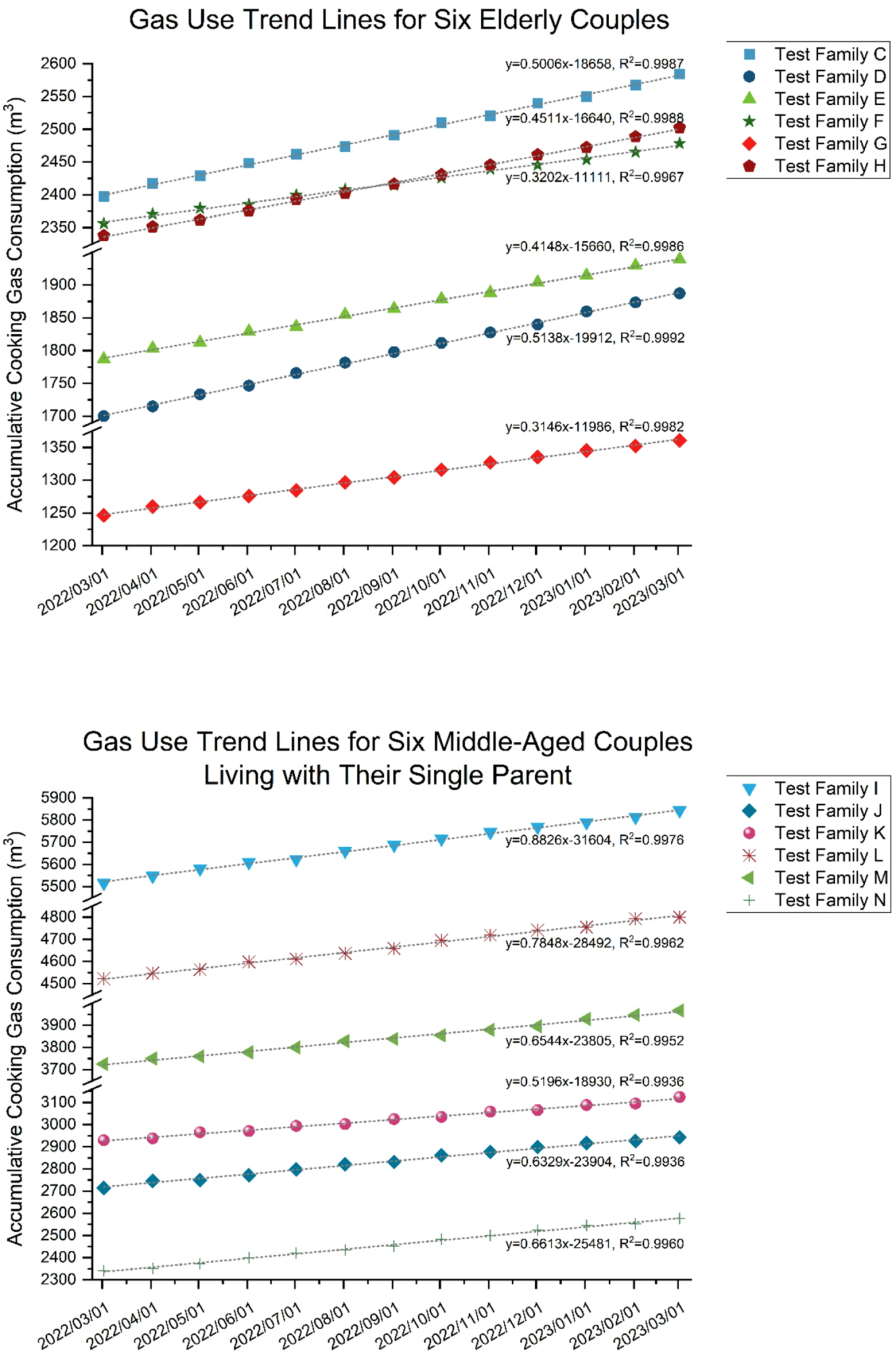
Gas use trendlines for two groups of test families.

For example, among the 40 responses, the two most common family structures were retired couples and middle-aged couples taking care of one of their parents, which are not uncommon in China. The gas usage from March 2022 to February 2023 for these two-family structure types is plotted in Fig. 4. By observing the graphs, it can be noted that the slopes representing the cooking energy use of these two-family structure types, respectively, exhibit strong similarities. The slopes for the six retired couples’ households range from 0.31 to 0.51, while the slopes for the six three-person households range from 0.52 to 0.88, with no overlap between the two groups. The remaining questionnaires also displayed similar clustering results for similar family structures.

The perspective of categorising family structures, in fact, aligns with the concept of the Family Life Cycle (FLC) presented in previous academic research. FLC is a statistical method originating in 1902^65^ that considers income, family size, demands, and ties with outside organisations changing over a family’s life cycle, following certain regularities^66–68^. This paper adjusts a previous FLC classification that is adapted to the Chinese family culture^69^, and integrates ten FLC categories into the experiment (Table 3).

The survey of 162 families regarding family members, gas consumption, time of eating at home, cooking utensils, food types, and cooking methods reveals the corresponding cooking energy consumption patterns related to FLC.

**Table 3:** Cooking energy consumption by stage of FLC.

Stage of FLC	Cooking energy (kWh/day)
Young couple, < 35 years old	Various
Middle-aged couple, 35 ~ 60 years old	5.1
Elderly couple, > 60 years old	4.13
A couple staying with their parents	6.8
Full nest I: young couple with dependent child, dependent child under 6	Various
Full nest II: young couple with dependent child, dependent child 6 ~ 12 years old	Various
Full nest III: middle-aged couple with dependent child, dependent child over 12	6.51
Key family I: three-generation family with the youngest dependent child under 6	12.86
Key family II: three-generation family with the youngest dependent child over 6	N.A.
Key family III: three-generation family, with a dependent child	8.13

The data show there is a strong relation between FLC and household energy, except for young couples, especially those with a child under 12, who exhibit uncertainty in their daily cooking habits due to frequent dining out. The survey highlights that in China, a family with three generations living together and the youngest child under 6 consumes the most cooking energy (12.9 kWh/day), while elderly retired couples consume the least (4.13 kWh/day).

A cooking energy lock-in effect was observed in our study, analogous to the well-known carbon lock-in phenomenon in energy and climate change contexts. Just as early choices in energy infrastructure can lock in a certain level of carbon emissions for decades, here the ingrained behaviours and needs of families at a given life cycle stage appear to lock in a typical level of cooking energy use (CookEUI) for that stage. For example, a young family with small children (Key Family I) may be “locked in” to high daily cooking energy because of the need to prepare fresh meals for the child and perhaps the presence of grandparents who prefer traditional cooking. On the other hand, an elderly couple may be locked into a routine of simple, frugal cooking, resulting in consistently lower energy use. These patterns remain relatively constant until the family transitions to a different life cycle stage (e.g. when children grow up and move out, etc.), at which point a new lock-in level might be established.

### Cooking behaviour contributing to the Lock-in effect

**Fig. 5:**
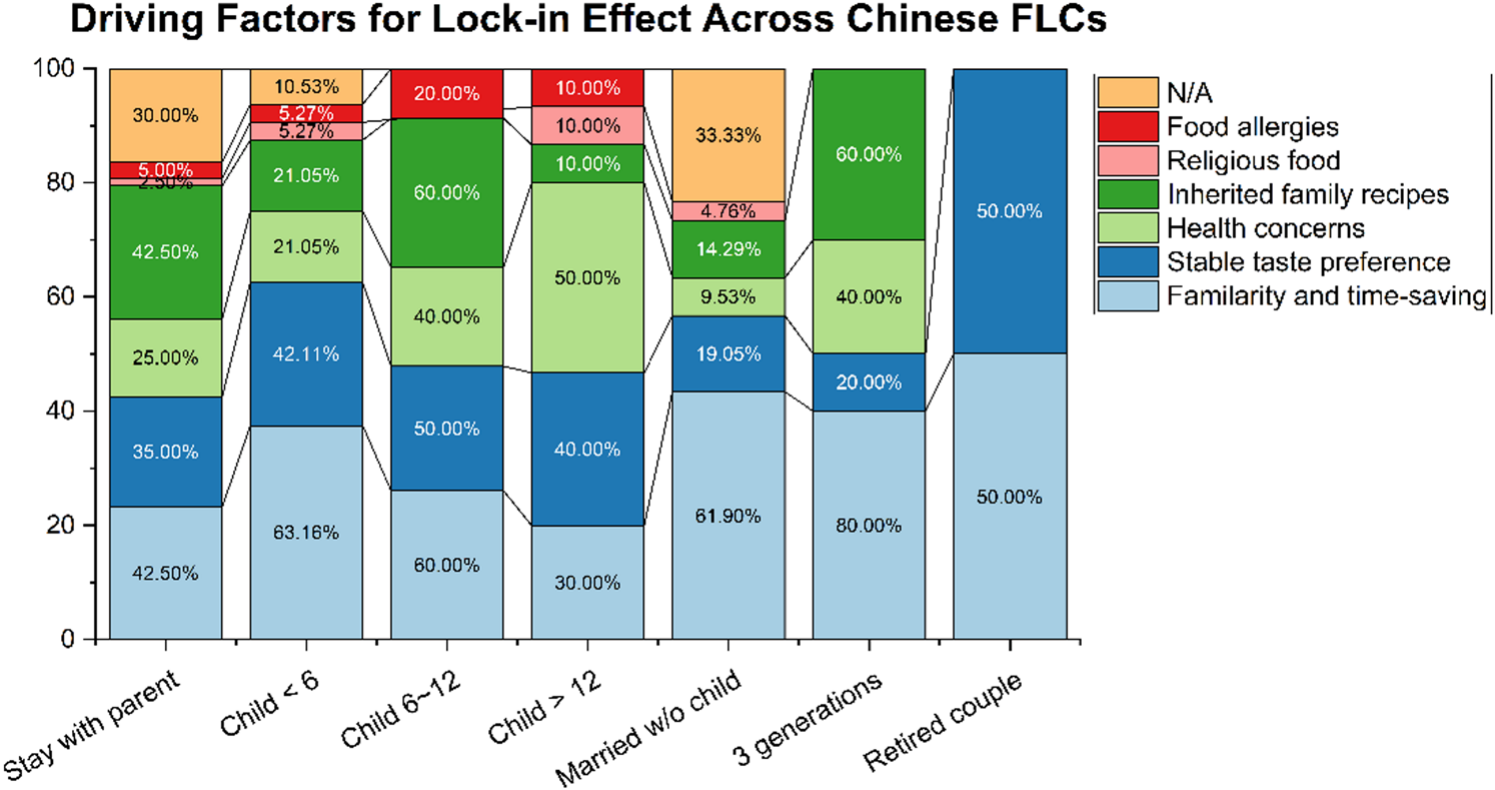
Driving factors for the lock-in effect across FLCs.

The lock-in effect is used here as an explanatory framing, and no causal claims are made. The driving factors of the lock-in effect between FLC and cooking energy were revealed by studying the 162 survey questionnaires. The survey indicates that the repetitive cooking of specific dishes results in this lock-in effect (Fig. 5). The primary reasons for repeatedly choosing certain dishes are that familiarity leads to less time for food preparation, followed by an unwillingness to change due to taste preferences. Respect for menus left by the older generation and health considerations are also key contributors. Additionally, 66% of the 162 questionnaires show that there is a fixed member who does the cooking, which is also a reason for the lock-in effect.

**Fig. 6:**
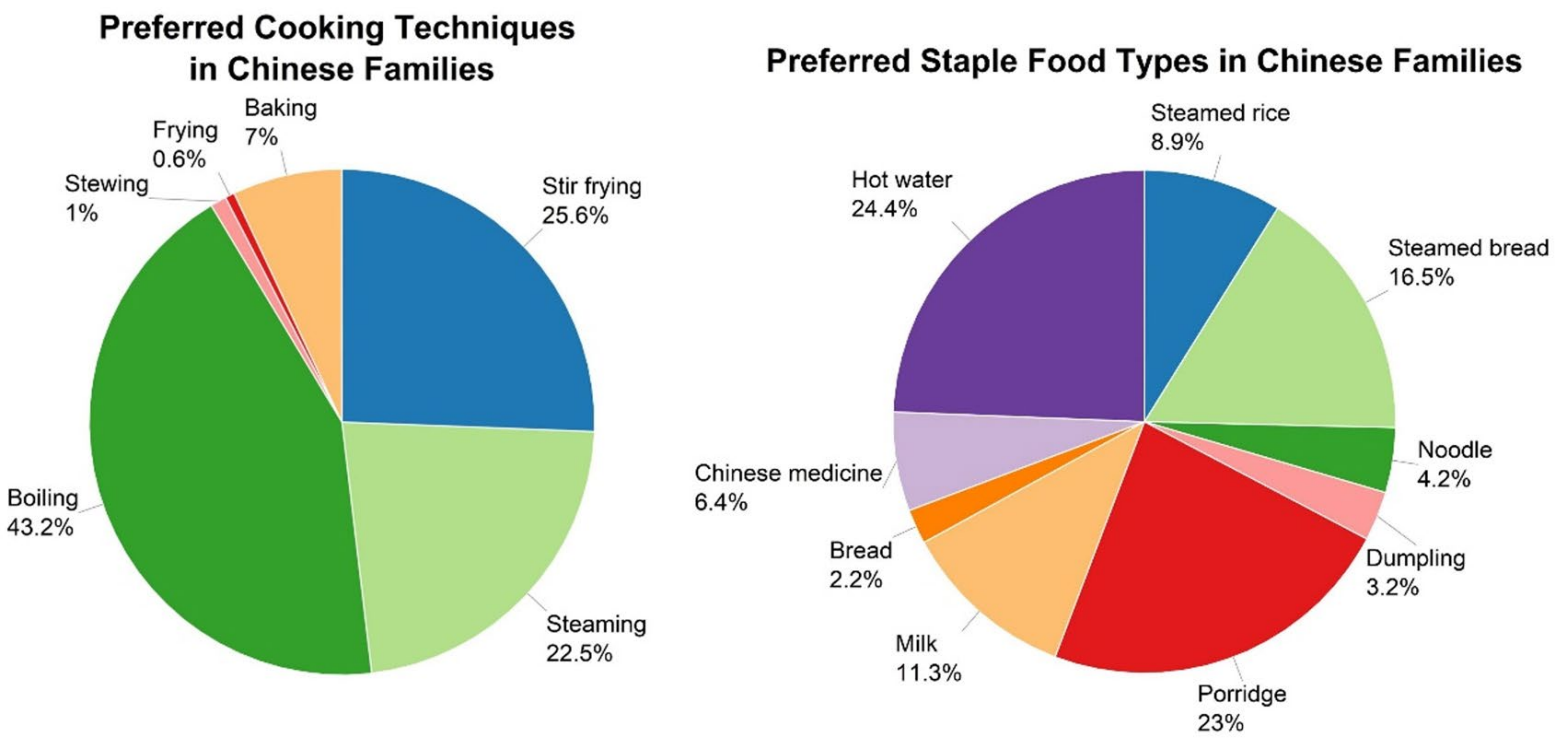
Preferred cooking techniques and staple food types in Chinese families.

Families at different FLC stages are also locked into distinct cooking techniques and staple food types. Boiling, steaming, and stir-frying are the top three cooking techniques applied in Chinese households, while hot water, porridge, and steamed foods are the most popular staple food types (Fig. 6).

**Fig. 7:**
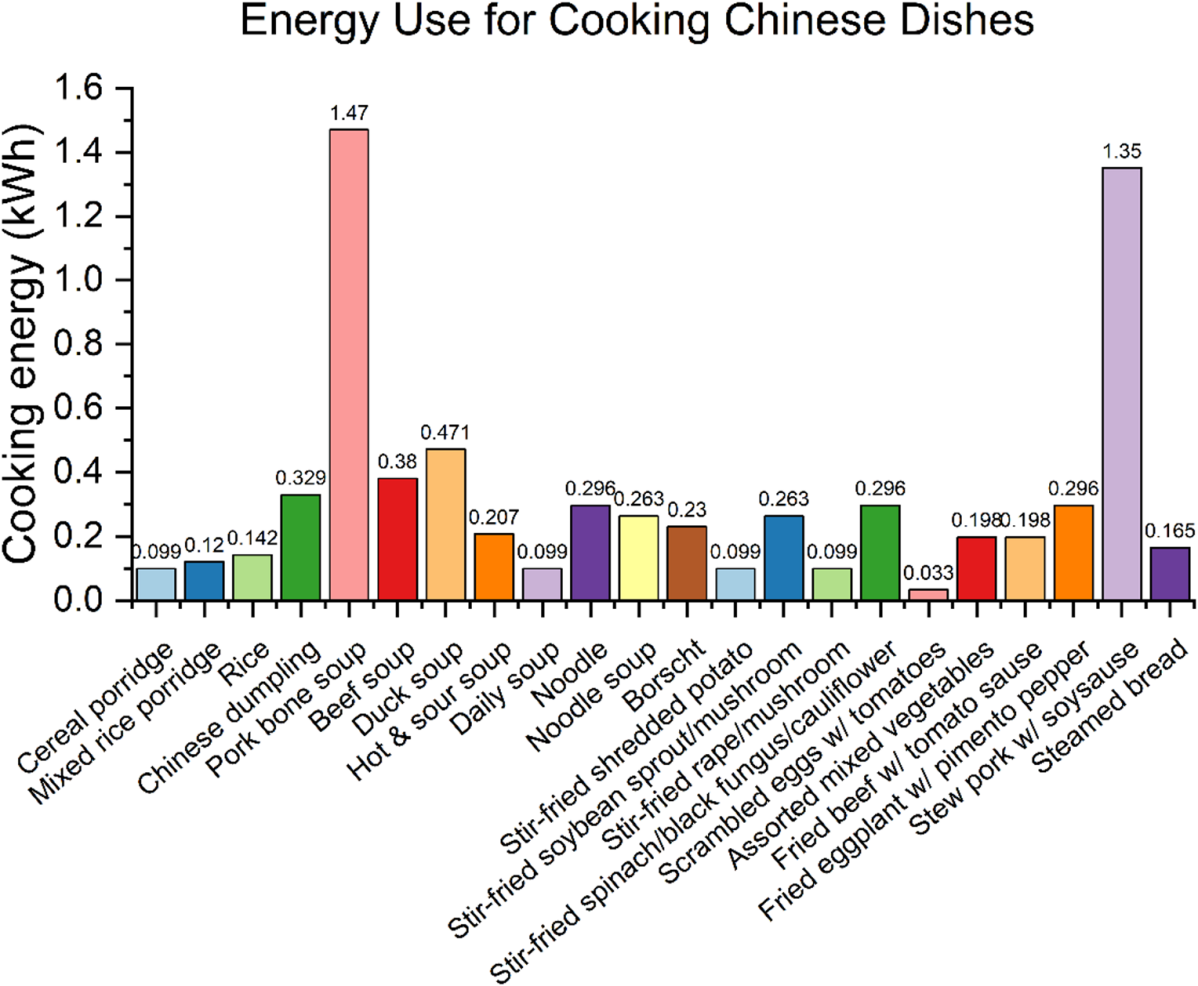
On-site cooking energy measurement for Chinese dishes.

These data illustrating Chinese food preferences for water boiling or steaming explain why Chinese families use more cooking energy than Western families. Water’s limited temperature (100 ˚C) compared to oil (over 200 ˚C) results in slower heat transfer to food, requiring more energy. Additionally, water’s higher heat capacity requires more energy to reach its boiling state. Figure 7 shows on-site measurements of energy used for cooking specific dishes that support this assumption: cooking energy use in China is higher compared with the tendency of Western countries to consume ready-to-eat or grilled and baked foods.

## Discussion

In-depth discussions of the experimental data yield several implications, offering guidance for future building energy simulations and real-world scientific research and development on cooking technologies in buildings.

### CookEUI versus traditional EUI

As outlined in the Introduction, our contributions are a household-normalised metric (CookEUI), descriptive FLC-to-CookEUI patterns, and simulation-ready inputs assignable by occupant profiles. In the building energy sector, Energy Use Intensity (EUI) in kWh/m^2^ is a common parameter for describing energy consumption. EUI has the advantage that it weakens the impact of different floor areas, allowing for the comparison of different building types worldwide at the same level^70^ and the prediction of a building’s annual energy consumption by multiplying the floor area by EUI benchmarks^19^.

This study has uncovered notable disparities in cooking energy consumption among residential units. Commonly used building energy simulation software like IESVE and eQUEST often generalises households, inaccurately categorising cooking energy as part of the process load and estimating it based on power intensity (W/m²). Such generalisations, however, overlook the significant role cooking energy plays in China’s overall building energy consumption, leading to substantial inaccuracies in energy consumption forecasts.

In response to this issue, we propose a more tailored metric: Cooking Energy Use Intensity (CookEUI), measured in kWh/day for a household (or normalised per household, rather than per floor area). The CookEUI concept directly correlates with family composition and life cycle stage, as evidenced by our data. By integrating CookEUI values into building energy simulations, practitioners can input specific proportions of different family types within a residential building (reflecting the building’s intended or typical occupants) to obtain more precise estimates of cooking energy demand. For example, if a new apartment building is expected to house a mix of young families, middle-aged families, and some elderly residents, the simulation could assign appropriate CookEUI values to those units instead of a one-size-fits-all cooking load. This approach would significantly enhance the accuracy of residential energy models for China by accounting for cultural and behavioural factors.

**Fig. 8:**
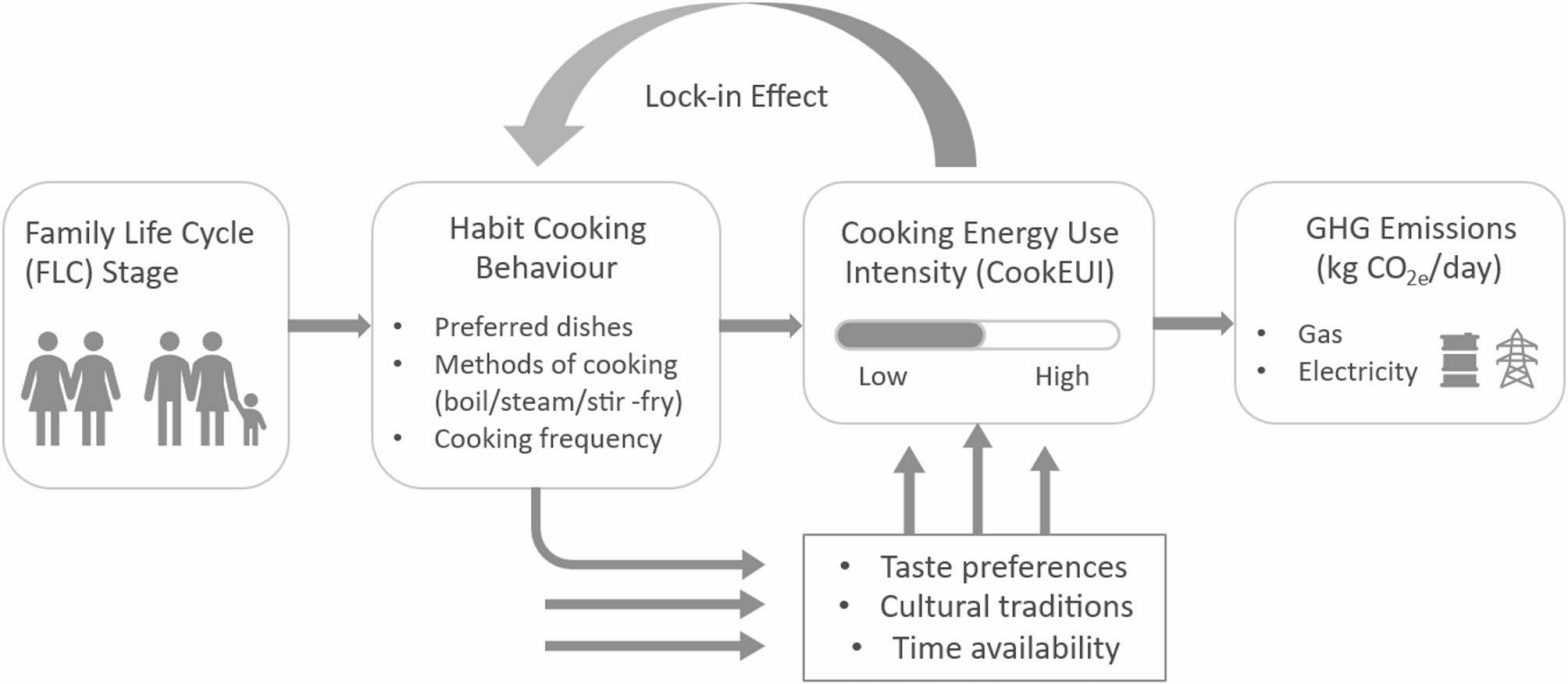
Mechanism linking FLC, cooking behaviour, and energy/emissions. (Note: the diagram is conceptual and descriptive; it does not imply causal identification)

The relationships among family life cycle, habitual cooking behaviour, and resulting energy use are illustrated conceptually in Fig. 8. In this framework, a family’s life cycle stage influences its cooking habits (preferred dishes, methods, and frequency of cooking), which in turn determine the daily cooking energy consumption (CookEUI) and the associated GHG emissions. Key underlying factors (such as time availability, taste preferences, and cultural traditions) drive the consistency of these cooking habits within each FLC stage, thereby locking in the energy intensity. The CookEUI metric encapsulates this locked-in intensity of cooking energy use that is characteristic of each family type. Conceptual note on lock-in: In this study, ‘lock-in’ is used as a conceptual lens linking FLC, habitual practices and CookEUI, consistent with prior household-energy literature cited here. Given our case-report design, this framing is descriptive only and bounded by sample size and context, and it does not imply causal or generalisable claims.

### The potential of cooking energy reduction

The observed lock-in effect of CookEUI with FLC, cooking behaviours, and food types has evolved over millennia. Cultural determinants make it unrealistic to expect significant energy reductions through behavioural change alone. Moreover, survey responses revealed that many households’ dietary choices are influenced by health concerns, allergies, and long-standing preferences. Some high heat-transfer media, such as oil, although energy-efficient, are restricted due to their health risks of fried foods^71^. Therefore, rather than altering deeply ingrained culinary traditions, technological innovation and energy transition present more feasible solutions to reducing cooking energy use. For instance, a pressurised cooker is an efficient alternative to traditional pots and kettles, increasing heating efficiency by increasing the pressure and raising water temperature beyond 100 ˚C. Studies show up to a 44% energy saving with a pressurised cooker compared to open cooking, with added benefits for food nutritional value^50,72^. Microwave ovens reduce energy consumption by 12–33% compared to conventional methods^73^.

In terms of energy sources, transitioning from natural gas to electricity is a low-carbon emission option. According to IPCC estimates, traditional gas stoves emit about 0.25 kg/person of CO_2_ per meal, whereas electric kitchens emit 0.19 kg/person^74^. The decreasing cost and expanding scale of renewable energy use in China also make electric power a more suitable option for building energy.

However, a significant challenge lies in human habits and preferences, particularly among professional chefs who prefer the traditional use of gas stoves^75,76^. Similar situations have been found in China, where there is a lot of opposition on websites promoting fully electrified kitchens, as people have learned from the evolution of Chinese culinary culture over thousands of years that open flame is a key factor in refining food taste^77^. A compromise, such as a “dual-use stove” allowing open flames for stir-frying and an induction cooker for simmering, can reduce carbon emissions without sacrificing taste. Some leading brands in China have already introduced such products.

Another suggestion involves centralising cooking methods, particularly steaming and boiling, in residential community canteens. This approach, involving the preparation of large quantities of food in a single, communal pot, has the potential to significantly reduce the energy consumption associated with cooking in individual households. Given the considerable impact of cooking energy on overall building energy consumption, strategies that reduce this specific type of energy use could have a substantial effect on decreasing building-wide energy demands.

In addition, food delivery and out-of-home dining are relevant factors. In many urban areas, the increasing availability of meal delivery and out-of-home dining plausibly lowers at-home cooking frequency, thereby reducing CookEUI for otherwise similar households. Our monitored dataset does not include an explicit delivery variable, so we note this as a scenario pathway. Future work will pair energy logging with simple behaviour diaries or app-based e-receipt tags to quantify this effect. This aligns with our survey note that young couples often display less stable at-home cooking due to frequent dining out ("Recommendations for future work" section).

Given its substantial contribution, reducing cooking energy consumption holds significant potential for decreasing overall building energy consumption.

### Recommendations for future work

This study is based on two monitored case households; findings are not generalisable and should be interpreted as descriptive case evidence. Despite the success of the experiments in revealing the high proportion of cooking energy and the lock-in effect, some limitations need addressing in future work.

Firstly, while summarising CookEUI for different FLCs (Table 2), the varied CookEUI of three FLC stages, i.e., young couples with or without children, suggests a need for further research into the relationship between the frequency of eating out and home cooking energy. Future work will expand the monitored sample to enable formal statistical testing and uncertainty quantification.

Additionally, as this experiment only collected data in China, applying CookEUI to building energy simulation software globally requires extensive worldwide measurements or surveys capturing diverse cultural and dietary habits. Therefore, future research should not only continue to investigate cooking energy patterns within residential buildings but also expand to other types of buildings, such as restaurants and food centres, to gain a more comprehensive understanding of cooking energy usage across different architectural settings.

Furthermore, personal location systems, rather than questionnaire surveys, are suggested for behavioural studies, since surveys may lead to inaccuracies, especially with elderly participants, whose memories become short and vague on many occasions. Installing real-time personal location systems in test families may be a solution, although it requires more substantial technical and financial support.

## Conclusion

This study brings household cooking energy into clear focus through two longitudinal case reports complemented by a broader survey. It delivers the stated objectives by formalising CookEUI, describing its conceptual linkage to FLC, and outlining simulation-ready inputs for practice. By introducing the descriptive metric Cooking Energy Use Intensity (CookEUI) and examining it alongside family life cycle (FLC) stages and habitual behaviours, we identify a cooking-energy lock-in where families in similar FLC stages tend to stabilise at characteristic daily cooking energy levels. In our empirical context, three-generation families exhibited the highest CookEUI (~ 12.9 kWh/day on average), while elderly couples showed the lowest (~ 4.13 kWh/day). These case-based patterns underscore how culture, time constraints, and taste preferences shape routine cooking practices and, in turn, daily energy demand—relationships that conventional floor-area EUI metrics cannot capture.

Translating measured energy to CO_2e_ confirms that routine cooking can form a meaningful share of a household’s operational carbon footprint. Although our estimates are scenario-based and reported as ranges, they demonstrate actionable opportunities for decarbonisation at the kitchen scale. Although behavioural refinements are unenforceable due to deeply rooted culinary traditions and distinctive lifestyles of individual households, innovative technology choices (e.g., pressure cookers, targeted electrification/induction where acceptable, or hybrid gas-induction stoves to preserve desired culinary performance) can reduce CookEUI and associated emissions while respecting culinary traditions. At the building and planning levels, using CookEUI as an input improves the realism of residential energy simulations and helps to size electrical infrastructure and ventilation systems for anticipated occupant profiles.

These conclusions are exploratory and non-generalizable by design. Limitations include the small number of instrumented households, non-identical observation windows, reliance on self-reported elements in the survey, and uncertainty in emission factors. Accordingly, our results should be interpreted as mechanistic evidence and baselines rather than population estimates. Future work should expand to stratified samples across FLC × region × climate, extend longitudinal monitoring to full-year windows for multiple family types, refine CO_2e_ factors with regional electricity mixes and gas properties, and pair energy tracking with ventilation and indoor air quality measurements to capture co-benefits. Randomised or quasi-experimental trials of behavioural/technological interventions, plus multi-country comparisons of CookEUI under different culinary cultures, would further test generalisability.

## Supplementary Information

Below is the link to the electronic supplementary material.

Supplementary Material 1

Supplementary Material 2

## Data Availability

The datasets used and/or analysed during the current study are available from the corresponding author on reasonable request.
