# Supplementary material for: A case study discovering lock-in effects of culinary culture and behaviours on cooking energy use in Chinese homes
Source: Sci Rep. 2026 Jan 29;16:6565. doi: 10.1038/s41598-026-35302-1 (PMC12909932; doi:10.1038/s41598-026-35302-1)
Supplement: Supplementary file 1 — Supplementary Material 1 [file 41598_2026_35302_MOESM1_ESM.docx]

**Appendix A - Explanation of Figures and Tables**

Figure 1 Floor plan of Test Family A with RFID system installed (left) and Test Family B (right). Figure 1 concludes two floor-plan diagrams. Left: RFID indoor tracking layout in a retired couple’s home. Blue concentric circles mark coverage of “Locators” placed in the kitchen, bedrooms, bathroom, and living areas. A “Reader” sits near the entrance; a “Workstation” (computer) in the study records data from wristband tags. When a person enters a room, the locator triggers the wristband and logs room, person, and time. Right: Another apartment plan labelled Children’s Room, Kitchen, Toilet, Guest Room, and Master Bedroom, with a black arrow. A red line shows an example path from the entrance to a bedroom. Together, the diagrams show how the system measures time spent in rooms, especially for cooking activities.

Figure 2. Energy Use Breakdown of Test Family A and B. Figure 2 describe the energy consumption breakdown for Test Family A and B, illustrating distinct profiles in annual and quarterly energy use. For Test Family A, the annual data shows that space heating is the dominant energy expense, accounting for 57.25% of the total, followed by cooking at 23.25%, and hot water at 8%. Other energy uses, including lighting, cooling, and entertainment, represent smaller fractions of 0.5%, 0.28%, and 5.06% respectively. Conversely, the quarterly data for Test Family B displays a significant portion of energy use attributed to space heating (30.21%) and cooking (47.71%), with the latter being the most substantial. The 'Others' category, likely encompassing miscellaneous household appliances, constitutes 8.6% of their energy use, a notable contrast to Family A's annual percentage. Additionally, lighting and hot water account for 4% and 4.67%, respectively, with cooling at a minimal 0.89%. This comparative visualization highlights the disparities in energy prioritization and usage between the two families over different time scales, offering insights into the variable impact of lifestyle choices on household energy profiles.

Figure 3. The accumulative cooking energy use of Test Family A (top) and Test Family B (bottom). Figure 3 illustrates the trend of cumulative cooking energy consumption for Test Family A (top graph) and Test Family B (bottom graph) over a specified time period. Both graphs plot cooking energy use in kilowatt-hours (kWh) on the y-axis against time on the x-axis, with data points corresponding to different dates. The linear trend lines, shown in red, represent the rate of increase in energy use over time, with their respective equations displayed. For Test Family A, the slope of the trend line is 6.3822, indicating a slower rate of increase in energy use over time. This is reflected in the less steep incline of the line. In contrast, Test Family B's trend line has a slope of 14.709x, suggesting a more rapid accumulation of energy consumption for cooking purposes. The equations for both trend lines include a 'y' for the cumulative energy use and an 'x' representing time. The intersect values (-245409 for Family A and -603542 for Family B) likely correspond to the starting points of the data collection relative to a particular baseline or starting date. These graphs are useful for comparing the long-term cooking energy consumption patterns of the two families, indicating how their cooking habits and energy use may differ over time. The data can provide insights into the effectiveness of energy-saving measures or the impact of lifestyle changes on energy consumption.

Figure 4. Gas use trendlines for two groups of test families. Figure 4 displays gas use trend lines for two distinct demographic groups of test families, represented over a series of dates. The top graph shows six elderly couples (Families C to H), and the bottom graph includes six middle-aged couples living with their single parent (Families I to N). Each family's gas consumption for cooking is tracked in cubic meters (m³) and plotted against specific dates spanning from March 2022 to March 2023, indicated by the format "yyyy/mm/dd." Different markers symbolize each family, and linear trend lines are fitted to their respective data points. Trendline equations and coefficients of determination (R-squared values) are shown for the trend lines. The slope coefficients of these lines (ranging from 0.3146x to 0.5138x for elderly couples, and from 0.5196x to 0.8826x for middle-aged couples with a single parent) represent the rate of increase in cooking gas consumption over time. The y-intercept values (ranging from -11986 to -19912 for elderly couples, and from -18930 to -31604 for middle-aged couples) indicate the initial values of cumulative gas consumption extrapolated back to a common starting point and reflect the length of each household's residence period. The trend lines illustrate the variation in cooking gas usage among the families, with middle-aged couples showing generally steeper slopes, indicating a faster rate of increase in consumption. This data can be instrumental in understanding household energy behaviours and developing targeted strategies for energy conservation within these demographic groups.

Figure 5. Driving factors for lock-in effect across FLCs. Figure 5 is a stacked column chart that presents the multiple-response survey results showing percentage of a group of driving factors for the cooking lock-in effect among seven distinct family types that represent different family life cycle (FLC) stages, namely singles or couples living with their parents, couple with a child aged under 6, couple with a child aged between 6 and 12, couples with a child aged over 12, married couple without a child, three generations living together, and retired couple. Within the column representing each family type, the chart shows the percentages breakdown of reasons why these families think they make relatively fixed cuisines. The response options include familiarity and timesaving, stable taste preference, health concerns, inherited family recipes, religious food, food allergies, and N/A (not applicable). From the figure, the most popular options are "familiarity and timesaving", "stable taste preference", "health concerns", and "inherited family recipes". The figure shows various reasons leading to the occurrence of cooking energy lock-in effect, demonstrates the robustness of this effect and supports the perspective that the change of cooking behaviours can be difficult to enforce.

Figure 6. Preferred cooking techniques and staple food types in Chinese families. Figure 5 juxtaposes the preferred cooking techniques and staple food types within Chinese families, illustrated in two pie charts. The chart on the left delineates cooking methods, with boiling being the most prevalent at 43.2%, followed by stir frying and steaming, accounting for 25.6% and 22.5% respectively. Baking constitutes 7%, while both frying and stewing are the least common, making up only 0.6% and 1%. The chart on the right details the staple food consumption patterns. Porridge holds a significant share at 23%, indicative of its staple status in Chinese diets. Steamed bread (mantou) is also a major component, representing 16.5%, while hot water, presumably for tea or other uses, comprises a considerable 24.4%. This reflects the cultural importance of hot beverages and soups. Steamed rice, a cornerstone in Chinese cuisine, accounts for 8.9%. Lesser consumed staples include milk at 11.3%, noodles at 4.2%, and dumplings at 3.2%. The chart also indicates a 6.4% preference for Chinese medicine, suggesting its integration into daily consumption habits. Together, these charts provide a comprehensive overview of the culinary practices in Chinese households, highlighting the diversity in cooking techniques and the prominence of certain foods and beverages in daily consumption. These preferences have implications for energy consumption patterns and nutritional habits in the context of Chinese family life.

Figure 7. On-site cooking energy measurement for Chinese dishes. Figure 6 provides an on-site cooking energy measurement for various Chinese dishes, depicted as a bar graph where the y-axis represents the energy consumption in kilowatt-hours (kWh). The dishes are arrayed along the x-axis and include a range of typical Chinese foods from cereals and porridges to meats and vegetables. The graph shows the highest energy consumption for 'Beef Soup' at 1.470 kWh, indicating it requires the most energy to prepare. Conversely, 'Scrambled Eggs with Tomato' and 'Stir-fried Spinach/Mushroom' are among the dishes with the lowest energy usage, at 0.033 kWh. The 'Steamed Bread' stands out with a significant consumption of 1.350 kWh, suggesting the energy-intensive nature of steaming this staple food. Most dishes fall within the lower energy consumption range, highlighting the efficiency of typical Chinese cooking techniques for everyday meals. The variation in cooking energy across dishes reflects the different cooking methods and durations required, providing insights into the potential energy savings that can be achieved by choosing less energy-intensive cooking options. The colour coding of the bars may indicate categories such as meats, vegetables, or staples, offering a visual differentiation of energy use by food type. This data is essential for understanding the energy footprint of culinary practices and can inform strategies to reduce energy consumption in Chinese cooking.

Figure 8. Mechanism linking FLC, cooking behaviour, and energy/emissions. Figure 7 is a schematic diagram illustrating the conceptual pathway of the cooking energy lock-in mechanism. The diagram consists of a sequence of three main boxes with arrows flowing from left to right, plus an outcome at the end: (1) Family Life Cycle (FLC) Stage – this leftmost box represents the family’s life stage category (young couple, family with children, retired couple, etc.). (2) Locked-in Cooking Behaviour – the middle box represents the characteristic cooking habits and practices that a family develops, strongly influenced by its FLC stage. Within this box, key drivers are noted (such as “preferred methods & repetitive dishes” and underlying factors like time-saving needs, taste preferences, and tradition). Essentially, a family’s life stage leads to certain habitual behaviours: for example, a busy young family might repeatedly cook quick dishes and rely on known recipes due to time constraints and established tastes, while an older couple might stick to a routine of simple traditional meals. (3) Cooking Energy Use Intensity (CookEUI) – the next box to the right indicates the outcome of those behaviours in terms of energy, i.e., a relatively fixed daily cooking energy requirement (in kWh/day) that is “locked in” for that family type. The diagram notes this as a “High (or characteristic) cooking energy use intensity locked to FLC.”

Table 1. Household energy use by category for Test Family A and B. Table 1 presents a detailed comparison of household energy consumption between Test Family A and Test Family B, divided into several categories. The data, measured in kilowatt-hours (kWh) for electricity and cubic meters (m³) for gas, provides insight into the energy use of specific home appliances and activities. For both families, energy consumption is categorized under entertainment electronics, refrigeration, hot water, washing machines, cooling, cooking appliances, other plug-in loads, lighting, and heating. Test Family A shows notably higher total electricity usage at 2172.00 kWh, compared to 683.29 kWh for Test Family B. This is seen across most categories, with disparities in the use of set-top boxes (STB) in bedrooms and dining/guest rooms, refrigerators, and water heaters. Cooking energy use is also higher for Family A, especially for electric rice cookers. However, Family B has increased energy usage for air conditioning in the dining/guest room. Other plug-in loads and lighting are higher for Family A, whereas Family B uses less energy in these categories. The table also equates gas usage to kWh based on a conversion factor provided by the State Administration of Market Regulation, indicating that Family A uses more gas as well. This comparison underscores the variability in household energy consumption based on appliance usage and lifestyle choices.

Table 2. Table 3 summarizes the cooking energy consumption and associated carbon emissions for the two case study families, providing a side-by-side comparison. For each family (A and B), it lists the monitoring period duration, the total cooking energy consumed in that period (in kWh).

Table 3. Cooking energy consumption by stage of FLC. Table 2 categorizes cooking energy consumption in kilowatt-hours per day (kWh/day) across different stages of the family life cycle (FLC). The FLC stages include young couples under 35 years, middle-aged couples between 35-60 years, and elderly couples over 60 years, showing varying daily cooking energy uses of 'Various', 5.1 kWh, and 4.13 kWh, respectively. For couples cohabiting with parents, energy consumption is significantly higher at 6.8 kWh/day. The table further distinguishes between families with dependent children, categorized as 'Full nest I' for those with children under 6 years, 'Full nest II' for children aged 6-12 years, and 'Full nest III' for middle-aged couples with children over 12 years. These categories are marked as 'Various' for the first two and 6.51 kWh for the third, indicating the change in cooking energy as children age. Additionally, three-generation families are classified as 'Key family I, II, III', with the youngest child's age impacting consumption. 'Key family I', with the youngest child under 6 years, has the highest consumption at 12.86 kWh/day. 'Key family II', with older dependents, does not have available data (N.A.). 'Key family III', regardless of the child's age, uses 8.13 kWh/day. This delineation reflects the influence of family composition and life stages on household energy use, with 'Various' indicating fluctuations or a range not specified.
