# Supplementary material for: A case study discovering lock-in effects of culinary culture and behaviours on cooking energy use in Chinese homes
Source: Sci Rep. 2026 Jan 29;16:6565. doi: 10.1038/s41598-026-35302-1 (PMC12909932; doi:10.1038/s41598-026-35302-1)
Supplement: Supplementary file 2 — Supplementary Material 2 [file 41598_2026_35302_MOESM2_ESM.docx]

**Appendix B – Explanation of Parameters in Measurement**

| **Symbol** | **Name** | **Definition / Meaning** | **Unit** |
| --- | --- | --- | --- |
| E_gas | Gas energy | Energy from cooking natural gas over the observation period | kWh |
| V_gas | Gas volume | Volume of natural gas used for cooking in the period | m³ |
| CF_gas | Gas-to-energy conversion factor | Converts gas volume to energy | kWh·m⁻³ |
| CookEUI | Cooking Energy Use Intensity | Average daily cooking energy of one household | kWh/day |
| E_cook,period | Cooking energy (period) | Total cooking energy in the observation period | kWh |
| E_elec,cook | Cooking electricity (period) | Electricity used by cooking appliances in the period | kWh |
| E_gas,cook | Cooking gas energy (period) | Gas energy used for cooking in the period | kWh |
| N_days | Number of days | Count of calendar days in the observation period | day |
| E_annual |  | Period energy converted to a yearly basis | kWh/year |
| E_period | Period energy | Energy for the chosen end-use over the period | kWh |
| CookEUI_annual | Annualized CookEUI | Daily CookEUI expressed per year | kWh/year |
| CO2e | Carbon dioxide equivalent | Total GHG emissions expressed as equivalent CO₂ | kg CO₂e |
| i∈{elec, gas} | Energy index | Index of energy carriers used for cooking | — |
| E_i | Energy by carrier i | Cooking energy for carrier i | kWh (elec) or m³ (gas) |
| EF_i | Emission factor for i | CO₂e per unit of energy (or volume) of carrier i | kg CO₂e/kWh or kg CO₂e/m³ |
| CO2e_day | Daily CO₂e | CO₂e from cooking per day | kg CO₂e/day |
| E_elec,day | Daily cooking electricity | Electricity used for cooking per day | kWh/day |
| EF_elec | Electricity emission factor | Emission factor for electricity | kg CO₂e/kWh |
| V_gas,day | Daily cooking gas volume | Gas volume used for cooking per day | m³/day |
| EF_gas,m³ | Gas emission factor (volume-based) | Emission factor per m³ of gas | kg CO₂e/m³ |
| EF_gas,kWh | Gas emission factor (energy-based) | Emission factor per kWh of gas energy | kg CO₂e/kWh |
| CO2e_year | Annual CO₂e | Annual cooking-related CO₂e | kg CO₂e/year |
| 365 | Days per year | Constant used for annualization | day |
